# An infant with persistent pulmonary hypertension of the newborn and congenital hypothyroidism

**DOI:** 10.1515/crpm-2026-0003

**Published:** 2026-09-11

**Authors:** Georgia Eva Fouli, Raghavendra Subba-Rao, Ravindra Bhat, Anne Greenough

**Affiliations:** Neonatal Intensive Care Unit, 4th Floor Golden Jubilee Wing, King’s College Hospital, London, UK; Department of Women and Children’s Health, School of Life Course Sciences, Faculty of Life Sciences and Medicine, King’s College London, London, UK

**Keywords:** persistent pulmonary hypertension of the newborn, hypothyroidism, inhaled nitric oxide

## Abstract

**Objectives:**

We report an infant who presented with persistent pulmonary hypertension of the newborn (PPHN) to highlight this was likely due to congenital hypothyroidism.

**Case presentation:**

A male infant born at term presented with respiratory distress and hypoxia two hours after birth requiring intubation and ventilation. Due to worsening clinical condition he was transferred from the local neonatal unit to a tertiary neonatal intensive care unit (NICU) for further management. An echocardiogram showed a structurally normal heart but with reduced right ventricular function and suprasystemic pulmonary artery pressures of 80 mmHg. There he required additionally inhaled nitric oxide, hydrocortisone, and intravenous fluid boluses. Sepsis was suspected as the underlying cause for the PPHN, but the infection screen was negative. He was subsequently diagnosed with congenital hypothyroidism on the newborn blood spot screening test. A technetium thyroid scan showed reduced uptake in the thyroid gland, in keeping with thyroid dyshormonogenesis. After consultation with the paediatric endocrinologists, he was commenced on levothyroxine and has made a good recovery.

**Conclusions:**

PPHN and congenital hypothyroidism have rarely been reported in association with each other. Yet, thyroid hormones play a crucial role in perinatal cardiovascular adaptation, through their effects on vascular resistance, cardiac contractility and response to circulating catecholamines. This case emphasises that it is important to fully investigate an infant with apparently unexplained PPHN for rarer causes.

## Introduction

Persistent pulmonary hypertension of the newborn (PPHN) has an incidence of approximately 2 per 1,000 births [1], 2]. Affected infants have hypoxaemia out of proportion to any chest radiographic abnormalities, with a pre- and post-ductal saturation difference of more than or equal to 10 %. PPHN can be classified into primary and secondary. Primary, or idiopathic PPHN is defined as normal pulmonary parenchyma with abnormal remodelling of the pulmonary vasculature. Secondary PPHN accounts for the majority of cases and can be classified into conditions affecting lung parenchyma, abnormal transition at birth and lung hypoplasia. Conditions affecting the lung parenchyma include meconium aspiration syndrome, sepsis as well as respiratory distress syndrome. Abnormal perinatal transition includes conditions such as transient tachypnoea of the newborn and congenital heart disease. Lung hypoplasia can be caused for example by premature prolonged rupture of membranes or congenital diaphragmatic hernia [3].

Treatment for PPHN is multifaceted and includes optimising oxygenation, ensuring haemodynamic stability and reducing pulmonary vasoconstriction. Furthermore, identification and treatment of the underlying cause is essential [2]. In severe cases, where the infant shows no response to treatment and demonstrates significant hypoxaemia, often with an oxygenation index of >40, extracorporeal membrane oxygenation (ECMO) can be considered [1], 2].

Thyroid hormone plays an important role in fetal development, including influencing cardiovascular system maturation, surfactant synthesis and brain maturation [4]. Congenital hypothyroidism, with a prevalence of 1:2,000–3,000 newborns is one of the most common causes of preventable intellectual disability in the UK. Early diagnosis is often made through the newborn blood spot screening test carried out on day five after birth. This is important as often symptoms do not present during the neonatal period, but early detection and treatment can lead to significant improvement in the child’s quality of life [5].

Causes of congenital hypothyroidism include maternal iodine insufficiency, thyroid gland dysgenesis or agenesis and occasionally thyroid dyshormonogenesis [4]. A review by Klosinska et al. 2022, further classified congenital hypothyroidism with a focus on preterm infants to include, primary (often due to abnormalities in thyroid development), permanent (due to dyshormonogenesis), transient (due to maternal antibodies or medications) and secondary or central (due to pituitary or hypothalamic abnormalities) [6].

We describe an infant who had pulmonary hypertension and congenital hypothyroidism and discuss the possible explanations for this association.

## Case presentation

A male infant was born at 38+0 weeks of gestation weighing 3,600 g. There was no significant family history, maternal virology was negative and maternal blood group was O positive. He was delivered via elective caesarean section as the baby was large for gestational age and the mother had polyhydramnios. There was no gestational diabetes mellitus. The infant was born in a district general hospital in good condition requiring no neonatal resuscitation immediately after delivery.

At two hours after birth the infant was admitted to the local neonatal unit due to increasing respiratory distress and hypoxia. He was started on high flow nasal cannula (HFNC) oxygen with fraction of inspired oxygen (FiO_2_) of 0.28. The initial chest radiograph showed signs of right sided consolidation and some bilateral haziness, but also oligaemia consistent with PPHN. Due to worsening respiratory acidosis and work of breathing, the infant was intubated and given surfactant at approximately 11 hours of age. The infant was started on broad spectrum intravenous antibiotics because of suspected sepsis and given intravenous fluids and a morphine infusion. He was also given a fluid bolus in view of hypotension with a mean arterial pressure (MAP) of 28 mmHg. Initial cardiovascular examination was normal with no pre and post ductal saturation difference. He was transferred to a local level two unit for further management.

During admission in the level two unit, he was noted to have persistent hypotension and was given two fluid boluses, started on a dopamine infusion at 15 μg/kg/min and received vecuronium. The infant was noted to have a fever of 38.4 °C and the initial CRP was 0.6 mg/L. Due to his worsening clinical condition, he was transferred to a tertiary centre for further management. Prior to transfer he had received a total of four fluid boluses, hydrocortisone and was treated for hypoglycaemia with an increase in the total fluid volume and dextrose concentration to 12.5 %. An initial echocardiogram showed a patent foramen ovale. An arterial blood gas result prior to transfer showed hypoxaemia with pH 7.461, pCO_2_ 3.29 kPa, pO_2_ 7.31 kPa, bicarbonate 21.0 mmol/L, base excess −4.1 mmol/L and lactate 3.1 mmol/L.

On admission to the tertiary NICU on day two, the infant was ventilated with synchronised intermittent mandatory ventilation (SIMV). Pre- and post-ductal saturations were 98 % and 96 % respectively. He was noted to have crackles bilaterally on auscultation of his lungs. The infant was started on dopamine and noradrenaline infusions. Antibiotics were continued and the admission temperature was 37.8 °C. An echocardiogram showed a structurally normal heart but with reduced right ventricular function and suprasystemic pulmonary artery pressures of 80 mmHg.

The infant was changed to continuous mechanical ventilation (CMV) on admission to the tertiary unit and started on inhaled nitric oxide (iNO) at a dose of 20 ppm. He was switched to patient triggered ventilation (PTV) with targeted tidal volume (TTV) on day three whilst sedation was weaned. iNO was continued until day five of life. The infant remained ventilated until day six and then remained on HFNC oxygen with an FiO_2_ of 0.35–0.4 until transfer back to the local hospital. Antibiotics were continued for a total seven days in view of rise in CRP to 79 mg/L. Blood culture and lumbar puncture culture were negative.

The infant was diagnosed with congenital hypothyroidism on the newborn blood spot screening on day 10. Initial thyroid stimulating hormone (TSH) and free thyroxine (T4) were 36.66 mIU/L (normal range 0.34–4.94 mIU/L) and 8.4 pmol/L (normal range 9.0–19.1 pmol/L) respectively. Technetium thyroid scan carried out on day 11 of life showed a normal location of the thyroid gland with reduced uptake in keeping with dyshormonogenesis. The results were discussed with the paediatric endocrinology and levothyroxine 35 mcg once daily was started. He had ongoing monitoring of thyroid function tests, with these normalising by day 43 of life.

## Discussion

The association between thyroid disorders and pulmonary hypertension has rarely been reported. One case report described a prematurely born infant with PPHN who also had poor growth, hypoglycaemia and cholestasis. The infant was later found to have hypopituitarism, adrenal and growth hormone insufficiency and euthyroid sick syndrome [7]. The PPHN resolved within two weeks of starting levothyroxine replacement. Another case report was of a baby with progressive respiratory failure and congenital hypothyroidism. A mutation was found in the *TTF-1/NKX2.1* gene which encodes the thyroid transcription factor 1 (TTF-1) protein. This protein is involved in lung, thyroid and central nervous development [8]. Both thyroid disorders and pulmonary hypertension are associated with mutations in the bone morphogenetic protein receptor type II (*BMPR2*) gene [9]. It should be noted, however, that the majority of those reports were in children and adults with pulmonary hypertension. In previous case reports, the infants were diagnosed with hypopituitarism and hypothyroidism respectively at several weeks old [7], 8]. This is in comparison to our case where congenital hypothyroidism was diagnosed on day 10 of life and treatment initiated almost immediately after diagnosis. It is thus likely that dyshormonogenesis would have almost certainly been present at birth and could have contributed to the presentation.

There are several possible explanations regarding the underlying mechanism of the association between PPHN and hypothyroidism. Thyroid hormones play a crucial role in the establishment of the newborn cardiovascular system. A surge in newborn TSH peaks at approximately 30 min after delivery. This is thought to be in response to cold exposure as well as catecholamine and cortisol release [10], 11]. The TSH level is also influenced by the mode of delivery and birth order [10]. This physiological increase in TSH leads to a rise in triiodothyronine (T3) and T4 [12]. Circulating fetal thyroid hormone levels pre-delivery play a crucial role in cardiovascular adaptation in terms of left ventricular function, heart rate and MAP [13]. Animal models with thyroidectomy during gestation, mimicking a hypothyroid state, do not have a surge in thyroid hormones after delivery. Those models have a lower left ventricular output, heart rate, MAP and oxygen consumption as well as lower blood flow to the peripheral circulation [13]. Levels of fetal thyroid hormone in the latter weeks of gestation effect organ development and adaptation to a greater extent than the surge of thyroid hormones that occurs postnatally [11], 13].

Thyroid hormones have been shown to alter vascular resistance, with a reduction in pulmonary vascular resistance noted during the postnatal thyroid hormone surge. [14]. One potential mechanism is through the effect of thyroid hormone on endothelin-1, a potent vasoconstrictor [14]. Animal models have shown that an increase in peripheral vascular resistance occurs in hypothyroidism and is corrected by a normalisation of thyroid hormone levels [15]. Studies have also assessed the effects of thyroid hormone on nitric oxide. They suggest that hypothyroid states reduce nitric oxide synthase activity and therefore reduce endothelium-dependent vasodilation [14], 16]. Thyroid hormones play a role in modifying cardiac contractility [11], 14]. This can be through their effects on influencing expression of proteins involved in cardiac contractility. Examples include altering cardiac myosin ATPase and proteins such as Ca-ATPase and Na-K-ATPase activity [11], 14]. Thyroid hormones also increase β1 adrenergic receptors on the myocardium thereby increasing cardiac response to sympathetic adrenal stimulation [11], 14].

In conclusion, we describe the rarely reported association of congenital hypothyroidism and PPHN. In this infant it is possible that sepsis also contributed to the PPHN, but blood and lumbar cultures were negative. This case report emphasises the importance of a thorough investigation of associations of PPHN particularly when common causes may have been eliminated.

## References

[1] Great Ormond Street Hospital (2016). Persistent pulmonary hypertension of the newborn (PPHN) [Internet]. ..

[2] Priya Cooper JP, Budhwani A, Kunneth LS, Patel IA, Omohundro A, Sholola OB (2025). Persistent pulmonary hypertension of the newborn. Prog Pediatr Cardiol.

[3] Singh Y, Lakshminrusimha S (2021). Pathophysiology and management of persistent pulmonary hypertension of the newborn. Clin Perinatol.

[4] Nallagonda S, Inusa A, Gupta R, Nallagonda M (2023). Thyroid disorders in neonates: a practical approach to congenital hypothyroidism and thyrotoxicosis. Paediatr Child Health.

[5] Great Ormond Street Hospital (2015). Congenital hypothyroidism [Internet]. ..

[6] Klosinska M, Kaczynska A, Ben-Skowronek I (2022). Congenital hypothyroidism in preterm newborns – the challenges of diagnostics and treatment: a review. Front Endocrinol.

[7] Pilgrim S, Stocker M (2007). Preterm infant with pulmonary hypertension and hypopituitarism SWISS SOCIETY OF NEONATOLOGY [Internet].

[8] Salerno T, Peca D, Menchini L, Schiavino A, Petreschi F, Occasi F (2013). Respiratory insufficiency in a newborn with congenital hypothyroidism due to a new mutation of TTF-1/NKX2.1 gene. Pediatr Pulmonol.

[9] Lecka-Ambroziak A, Kot K (2024). Pulmonary hypertension and hypothyroidism – still an important clinical coincidence in paediatric population, an endocrinologist’s point of view. Life.

[10] Lee SY (2016). Perinatal factors associated with neonatal thyroid-stimulating hormone in normal newborns. Ann Pediatr Endocrinol Metabol.

[11] Kotsopoulou I, Vyas AK, Cory MJ, Chan CS, Jagarapu J, Gill S (2022). Developmental changes of the fetal and neonatal thyroid gland and functional consequences on the cardiovascular system. J Perinatol.

[12] Doherty TM, Salik I, Hu A (2020). Physiology, neonatal [Internet].

[13] Breall JA, Rudolph AM, Heymann MA (1984). Role of thyroid hormone in postnatal circulatory and metabolic adjustments. J Clin Investig.

[14] Portman MA (2008). Thyroid hormone regulation of perinatal cardiovascular function. Semin Perinatol.

[15] Diekman M, Harms M, Endert E, Wieling W, Wiersinga W (2001). Endocrine factors related to changes in total peripheral vascular resistance after treatment of thyrotoxic and hypothyroid patients. Eur J Endocrinol.

[16] McAllister RM, Albarracin I, Price EM, Smith TK, Turk JR, Wyatt KD (2005). Thyroid status and nitric oxide in rat arterial vessels. J Endocrinol.

